# Occurrence and Characteristics of *Escherichia albertii* in Wild Birds and Poultry Flocks in Switzerland

**DOI:** 10.3390/microorganisms10112265

**Published:** 2022-11-15

**Authors:** Karen Barmettler, Michael Biggel, Andrea Treier, Francis Muchaamba, Barbara Renate Vogler, Roger Stephan

**Affiliations:** 1 Institute for Food Safety and Hygiene, Vetsuisse Faculty, University of Zurich, 8057 Zurich, Switzerland; 2 National Reference Centre for Poultry and Rabbit Diseases (NRGK), Institute of Food Safety and Hygiene, Vetsuisse Faculty, University of Zurich, 8057 Zurich, Switzerland

**Keywords:** *E. albertii*, wild birds, poultry, strain characteristics, WGS

## Abstract

*Escherichia albertii*, a zoonotic pathogen, has sporadically been associated with infectious diarrhea in humans. Poultry and wild birds are considered potential reservoirs. We assessed the occurrence of *E. albertii* in 280 fecal samples from wild birds (*n* = 130) and pooled fecal samples collected at slaughterhouse level from poultry flocks (*n* = 150) in Switzerland. Using an *E. albertii*-specific PCR targeting the *Eacdt* gene, 23.8% (31/130) of the samples from wild birds, but not from the pooled poultry fecal samples, tested positive for *Eacdt*. The positive samples originated from 11 bird species belonging to eight families. Strain isolation was attempted on the PCR-positive samples by subculturing the broth cultures onto xylose–MacConkey plates. Isolation was possible on 12 of the 31 *Eacdt*-PCR-positive samples. Whole-genome sequencing revealed that the strains belonged to nine distinct sequence types, with ST13420 and ST5967 being represented by two and three isolates, respectively. All strains harbored the *eae* gene, while two strains were also positive for *stx2f*. Our study thus shows that *E. albertii* is present in the Swiss wild bird population, which can potentially act as a source of this pathogen to humans, other animals, and the environment.

## 1. Introduction

*Escherichia albertii* is a close relative of *E. coli* and has sporadically been associated with infectious diarrhea and foodborne outbreaks [1,2,3,4,5,6]. It was first described as an atypical *eae*-positive *Hafnia alvei* isolated in 1991 from a diarrheic child in Bangladesh [7], and was reclassified in 2003 as a new taxon in the genus *Escherichia* [8]. Due to its genetic and phenotypic similarity to other Enterobacteriaceae and the presence of *eae*, a virulence gene typically associated with enteropathogenic *E. coli* (EPEC) and enterohemorrhagic *E. coli* (EHEC), *E. albertii* is often misidentified in routine diagnostics [6,7,9,10,11,12,13,14,15,16].

In humans, clinical signs of *E. albertii* infection resemble those of a typical bacterial enteric infection, consisting of watery diarrhoea, nausea, vomiting, fever, abdominal pain, dehydration, and bloating [6,7,17]. Disease manifestations are linked to intestinal lesions and result from the combined action of intimin, an *eae* gene-encoded outer membrane protein, and type III secretion system effectors—similar to those caused by EHEC and EPEC [18,19,20]. Animals infected with *E. albertii* are often subclinically infected or die acutely with pathologic findings of septicaemia [18,21]. 

Some *E. albertii* strains have been reported to produce Shiga toxin 2a (*Stx2a*) or 2f (*stx2f*) [11,12,19,22,23]. Its clinical significance is not yet fully understood, but should not be underestimated, as Shiga toxin is the primary virulence factor of EHEC, and *stx*-positive *E. albertii* have sporadically been associated with bloody diarrhoea [24] and haemolytic uremic syndrome [25]. The virulence factor *Stx2a* is frequently associated with complicated infections and severe symptoms in humans, while *stx2f* has not yet been detected in severely ill patients. The clinical significance of *stx2f*-producing *E. albertii* is therefore still unknown [24,26]. Another important virulence factor is the cytolethal distending toxin (*cdt*). This toxin consists of three subunits (CdtA, CdtB, and CdtC) and is encoded in the *Eacdt* gene, which is highly specific for *E. albertii* [27]. The subunit CdtB acts as a DNAse and leads to DNA double-strand breaks, which in turn leads to irreversible cell cycle arrest [11]. The factor CDT has been associated with persistent colonization and invasion [28,29]. Wild-type *E. albertii* are susceptible to clinically relevant antibiotics, but the emergence of multi-drug-resistant clones has been reported [30]. 

A recently published up-to-date overview on the importance and occurrence of *E. albertii* is available [27]. In previous studies, birds have been described as potential carriers of *E. albertii* that might act as infection sources for humans [18,31,32,33]. Poultry were identified as a major source of multi-drug-resistant *E. albertii* in China [30]. In this study, we screened birds in Switzerland as potential carriers for *E. albertii* to assess the threat posed to people routinely handling these animals. Isolates were confirmed as *E. albertii* by *Eacdt*-specific PCR and further characterised by whole-genome sequencing.

## 2. Materials and Methods

### 2.1. Sample Collection

Fecal samples were collected between March and August 2022 from (1) avian patients at the rehabilitation center of the Swiss Ornithological Institute, (2) avian patients at the “Greifvogelstation Berg am Irchel”, (3) dead/injured birds collected by a gamekeeper of the city of Zurich, and (4) broiler flocks at a slaughterhouse. Overall, 280 samples were collected, representing 26 species from 13 orders (Table 1). Samples were taken by swabs (Transwab Amies sterile, with Amies medium MW170; HuberLab), using freshly defecated feces, or, in the case of dead birds, from the cloaca. For broilers, samples were taken at the slaughterhouse level from feces deposited in transport cages and pooled by flock. Samples from 150 flocks were collected, each consisting of 1921 to 17,513 birds (in total, 1,127,276 broilers). For all sampled wild birds, species, site of sample collection, date, age, sex, any clinical findings, cause of death, and as available, patient file number were recorded.

### 2.2. Bacterial Enrichement, Growth Conditions, and DNA Extraction

All swab samples were enriched in *Enterobacteriaceae* enrichment (EE) broth (Becton, Dickinson, Heidelberg, Germany) and incubated at 42 °C overnight. A loopful of each of the enrichment cultures was then plated on sheep blood agar (DifcoTM Columbia Blood Agar Base EH; Becton Dickinson AG, Allschwil, Switzerland), and again incubated at 42 °C overnight. The colonies were subsequently washed off with 2 mL 0.85% NaCl solution. Of this colony suspension, an aliquot (100 μL) was combined with 200 μL Gram-negative lysis buffer and heated at 60 °C for 50 min, followed by 99 °C for 10 min. After centrifugation (2 min; 11,000 rpm), the supernatant was used as template for the PCR.

### 2.3. Screening for the Eacdt Gene

All samples were tested for the presence of the cytolethal distending toxin (*Eacdt*) gene. The PCR was performed as previously described [34] with slight modifications, using GoTaq^®^ Green Master Mix (Promega, Madison, WI, USA). The primer set EaCDTsp-F2 and EaCDTsp-R2 was used to specifically amplify a 449 bp fragment of the *Eacdt* gene. The *E. albertii* strain DSM 17528 was used as positive control, purified water as negative control. Amplification was done using the PCR thermocycler with cycle conditions of 5 min at 94 °C, followed by 30 cycles of 30 s at 94 °C, 30 s at 50 °C, 40 s at 72 °C, and lastly, 7 min at 72 °C. Results were analyzed using the Molecular Imager^®^ Gel Doc™ XR System.

### 2.4. Isolation of E. albertii

In the event of an *Eacdt*-positive PCR result, one loopful of each of the respective colony suspensions was streaked onto xylose–MacConkey plates [35] and incubated at 42 °C overnight. Unlike *E. coli*, most *E. albertii* cannot ferment xylose, and colonies appear colorless to grey-white. These suspicious colonies were then confirmed as colonies of *E. albertii* by retesting them for the presence of *Eacdt* by the PCR described above. One positive colony per sample was stored in 25% glycerol at −80 °C for the strain collection bank.

### 2.5. DNA Extraction and Whole-Genome Sequencing (WGS)

For DNA extraction, the strains were grown on sheep blood agar at 42 °C overnight. DNA was isolated using the DNA Blood and Tissue Kit (Qiagen, Hombrechtikon, Switzerland), and DNA libraries were prepared using the Nextera DNA Flex Sample Preparation Kit (Illumina, San Diego, CA, USA). Whole-genome sequencing was performed using Illumina MiniSeq (Illumina, San Diego, CA, USA). Illumina read quality was assessed using FastQC 0.11.7 (Babraham Bioinformatics, Cambridge, UK), and genomes assembled with SPAdes 3.14.1 implemented in shovill 1.1.1 [36,37] using default settings. Multi-locus sequence types (STs) were determined using PubMLST (https://pubmlst.org/) and the *Escherichia* spp. Achtman scheme [38]. Acquired resistance genes were identified using AMRfinder 3.10.24 (default parameters) [39]. Virulence genes were identified using ABRicate 1.0.1 (https://github.com/tseemann/abricate) in combination with the Virulence Factor Database set B (sequence coverage 70%, identity 90%) [40]. The intimin encoding *eae* gene was typed using ABRicate 1.0.1 (sequence coverage 70%, identity 97%) in combination with the *eae* database provided by Luo et al. [30]. Subtypes of *cdtB* were identified using ABRicate 1.0.1 (sequence coverage 70%, identity 97%) with nucleotide sequences of accession numbers AAD10622 (*cdtB*-I), AAA18786 (*cdtB*-II), AAC45443 (*cdtB*-III/V), AAT92048 (*cdtB*-IV), and AST83_RS10865 (*cdtB*-VI) as references. *E. albertii* O and H antigen genotypes were determined by in silico PCR (https://github.com/egonozer/in_silico_pcr) in combination with primer pairs described by Ooka et al. [41] and Nakae et al. [42]. Only exact matches were considered. SNP distances were determined by read mapping using the CFSAN SNP Pipeline 2.2.1 for each ST cluster separately [43].

## 3. Results

Overall, 31 of 280 samples (11.1%) were PCR-positive for *Eacdt* (Table 1). The highest prevalence rates were found in yellow-legged gulls (*Larus michahellis*) (4/4, 100%) and carrion crows (*Corvus corone*) (15/24, 62.5%). Other positive samples originated from cormorant (*Phalacrocorax carbo*) (*n* = 1/1), magpie (*Pica pica*) (*n* = 2/5), red kite (*Milvus milvus*) (*n* = 1/3), sparrowhawk (*Accipiter nisus*) (*n* = 1/3), mallard (*Anas platyrhynchos*) (*n* = 1/4), white stork (*Ciconia ciconia*) (*n* = 1/4), brown owl (*Strix aluca*) (*n* = 1/7), common buzzard (*Buteo buteo*) (*n* = 3/23), and common kestrel (*Falco tinnunculus*) (*n* = 1/18). All poultry samples were PCR-negative.

By streaking colony suspensions onto xylose–MacConkey plates, *E. albertii* isolates could be recovered from 12 (38.7%) out of the 31 PCR-positive samples and were available for whole-genome sequencing. These originated from red kite (*Milvus milvus*) (*n* = 1/1), sparrowhawk (*Accipiter nisus*) (*n* = 1/1), magpie (*Pica pica*) (*n* = 1/2), yellow-legged gull (*Larus michahellis*) (*n* = 2/4), carrion crow (*Corvus corone*) (*n* = 6/15), and common buzzard (*Buteo buteo*) (*n* = 1/3) (Table 1).

Ribosomal MLST typing confirmed an *E. albertii* species affiliation for all 12 whole-genome sequenced isolates. They belonged to nine distinct sequence types, with ST13420 and ST5967 being represented by two and three isolates, respectively. The two ST13420 isolates differed by only one SNP and originated from carrion crows that were kept at the same rehabilitation facility. Similarly, two (KBV63i and KBV86i) of the three ST5967 isolates differed by one SNP, with the third isolate being more distantly related (~95 SNPs). KBV86i originated from a wild carrion crow, while KBV63i was obtained from an injured common buzzard at a rehabilitation facility.

All 12 isolates harbored *eae* genes, most of which were identical or near-identical to known variants (Table 2). The ST13420 isolates possessed a novel *eae* variant (96.0% sequence identity with *eae* alpha8). Two isolates (KBV38i and KBV70i) contained *stx2f*. All isolates harbored the *cdtB* subtype II, and the two *stx2f*-positive isolates additionally carried *cdtB*-I in a second copy of the *cdtABC* operon. In silico PCR assigned most isolates to one of five distinct O-antigen genotypes (EAOg4, EAOg12, EAOg16, EAOg32, and EAOg36). Three isolates carried putative novel variants. None of the 12 isolates harbored acquired antimicrobial resistance genes. All isolates harbored a chromosomal *ampC* gene encoding an intrinsic beta-lactamase.

## 4. Discussion

*E. albertii* is frequently found in birds. So far, *E. albertii* has been detected in birds in Scotland, North America, Australia, Japan, and Korea. It has been isolated from 29 species belonging to 22 families: Anatidae, Ardeidae, Artamidae, Cacatuidae, Columbidae, Corvidae, Falconidae, Fringillidae, Hirundinidae, Maluridae, Meliphagidae, Motacillidae, Passeridae, Phalacrocoracidae, Phasianidae, Picidae, Procellariidae, Psittacidae, Pycnonotidae, Rallidae, Rhipiduridae, and Sturnidae [18,21,32,33,44]. In this study, we examined a total of 280 fecal samples from 26 bird species for the presence of the *Eacdt* gene by PCR.

We were able to detect putative *E. albertii* in 11 species belonging to eight families: Accipitridae, Anatidae, Ciconiidae, Corvidae, Falconidae, Laridae, Phalacrocoracidae, and Strigidae. Four of the eleven species are waterbirds. Considering that *E. albertii* has been detected in environmental samples such as water [15,45,46], a transmission via water is conceivable and possibly involves other wild animals. Most of the other seven species, including birds of prey and corvidae, have in common that they mainly inhabit farmland and forests and that they feed on birds and/or small mammals. Considering that small mammals are often carriers of pathogens, it cannot be excluded that they might also be a reservoir for *E. albertii*. However, data on this hypothesis are so far lacking.

Previous studies found a variable prevalence of *E. albertii* in poultry, poultry meat, and giblets [1,13,30,47,48,49,50]. In this study, pooled fecal samples of 150 broiler flocks representing more than one million birds were investigated. We could not detect *E. albertii* in any of these samples, demonstrating that poultry is not a primary reservoir for *E. albertii* in Switzerland. Further investigations should focus on laying hens, as they are more often housed outdoors and may thus get infected through contact with wild birds.

Several studies have reported challenges in isolating *E. albertii* from PCR-positive samples [35,46,48,51]. Hinenoya et al. [52] reported a recovery rate of 25% in a study on the presence of *E. albertii* in raccoons. In addition, for stool from healthy humans, a detection limit of 10^5^ CFU/g stool was reported [35]. In our study, the recovery rate was 38.7% (12/31). Other species such as *Shigella boydii* or *Providencia stuartii* are morphologically indistinguishable from *E. albertii* on the utilized selective medium, complicating the recovery of *E. albertii* [53]. Improved selective media are therefore needed to enable comprehensive epidemiological and clinical investigations of *E. albertii*. Alternatively, since we only considered colorless to grey-white colonies as suspicious, we might have missed lactose- or xylose-fermenting *E. albertii*, respectively, since these would not grow colorless [27]. Genome analyses of the 12 successfully recovered isolates identified two SNP clusters comprising two isolates each. One cluster was linked to carrion crows kept in the same rescue center, suggesting a recent transmission or acquisition from the same source, such as animal-to-animal spread or contaminated feed or water. The second cluster comprised isolates from a wild carrion crow and a buzzard from a bird sanctuary, pointing towards environmental transmission or indicating colonized prey as a potential source.

A sampling bias might exist for the wild birds since ill, injured, and dead birds were sampled. However, none of these birds showed clinical signs of intestinal infection, suggesting asymptomatic carriage. In conclusion, our study reveals that *Escherichia albertii* is present in the Swiss wild bird population, which can potentially act as a source of this pathogen to humans, other animals, and the environment.

## Figures and Tables

**Table 1:** List of sampled birds; number of *Eacdt*-positive samples and number of recovered *E. albertii* isolates.

Order	Family	Species	No. Specimens	No. (%) *Eacdt*-PCR Positive	No. *E. albertii* Isolates
*Accipitriformes*	*Accipitridae*	Black kite (*Milvus migrans*)	3	0	0
		Common buzzard (*Buteo buteo*)	23	3 (13.0%)	1
		European honey buzzard (*Pernis apivorus*)	1	0	0
		Red kite (*Milvus milvus*)	3	1 (33.3%)	1
		Sparrowhawk (*Accipiter nisus*)	3	1 (33.3%)	1
	*Pandionidae*	Osprey (*Pandion haliaetus*)	1	0	0
*Anseriformes*	*Anatidae*	Mallard (*Anas platyrhynchos*)	4	1 (25%)	0
*Charadriiformes*	*Laridae*	Yellow-legged gull (*Larus michahellis*)	4	4 (100%)	2
*Ciconiiformes*	*Ciconiidae*	White stork (*Ciconia ciconia*)	4	1 (25%)	0
*Columbiformes*	*Columbidae*	Common wood pigeon (*Columba palumbus*)	1	0	0
		Feral pigeon (*Columba livia domestica*)	14	0	0
*Falconiformes*	*Falconidae*	Common kestrel (*Falco tinnunculus*)	18	1 (5.6%)	0
*Galliformes*	*Phasianidae*	Broiler (*Gallus gallus domesticus*)	150	0	0
*Gruiformes*	*Rallidae*	Coot (*Fulica atra*)	1	0	0
*Passeriformes*	*Turdidae*	Blackbird (*Turdus merula*)	2	0	0
	*Passeridae*	House sparrow (*Passer domesticus*)	1	0	0
	*Corvidae*	Carrion crow (*Corvus corone*)	24	15 (62.5%)	6
		Eurasian Jay (*Garrulus glandarius)*	1	0	0
		Magpie (*Pica pica*)	5	2 (40%)	1
		Rook (*Corvus frugilegus*)	2	0	0
*Pelecaniformes*	*Ardeidae*	Gray heron (*Ardea cinerea*)	1	0	0
*Podicipediformes*	*Podicipedidae*	Great crested grebe (*Podiceps cristatus*)	3	0	0
*Strigiformes*	*Strigidae*	Brown owl (*Strix aluco*)	7	1 (14.3%)	0
		Eagle owl (*Bubo bubo*)	2	0	0
		Long-eared owl (*Asio otus*)	1	0	0
*Suliformes*	*Phalacrocoracidae*	Cormorant (*Phalacrocorax carbo*)	1	1 (100%)	0
Total			280	31 (11.1%)	12

**Table 2:** Sequence types and presence of *stx, eae* subtype, *cdtB* subtype, and *O*- and *H*-antigen subtypes in *E. albertii* genome assemblies.

Isolate	Source	Facility	MLST	*stx* Subtype	*eae* Subtype (Sequence Identity) *	*cdtB* Subtype	*O*-Antigen Genotype	*H*-Antigen Genotype	Accession Number
KBV4i	Carrion crow	RSOI	ST13420	-	Novel	*cdtB*-II	EAOg36	EAHg1	GCA_025600035.1
KBV24i	Carrion crow	RSOI	ST13420	-	Novel	*cdtB*-II	EAOg36	EAHg1	GCA_025599995.1
KBV30i	Carrion crow	RSOI	ST5967	-	sigma2 (99.9%)	*cdtB*-II	EAOg36	EAHg2	GCA_025599955.1
KBV26i	Magpie	RSOI	ST5399	-	sigma2 (99.96%)	*cdtB*-II	EAOg32	EAHg2	GCA_025599965.1
KBV27i	Carrion crow	RSOI	ST4685	-	alpha9 (100%)	*cdtB*-II	EAOg12	EAHg1	GCA_025599935.1
KBV38i	Yellow-legged gull	RSOI	ST8692	*stx2f*	xi (99.97%)	*cdtB*-I, *cdtB*-II	Novel	EAHg3	GCA_025599905.1
KBV42i	Carrion crow	RSOI	ST7834	-	not determined (incompletely assembled)	*cdtB*-II	Novel	EAHg1	GCA_025599895.1
KBV63i	Common buzzard	GBI	ST5967	-	sigma2 (99.9%)	*cdtB*-II	EAOg36	EAHg2	GCA_025599875.1
KBV70i	Sparrowhawk	GBI	ST11471	*stx2f*	sigma (100%)	*cdtB*-I, *cdtB*-II	EAOg4	EAHg4	GCA_025599835.1
KBV72i	Red kite	GBI	ST4170	-	alpha8 (100%)	*cdtB*-II	EAOg16	EAHg1	GCA_025599845.1
KBV86i	Carrion crow	E	ST5967	-	sigma2 (99.9%)	*cdtB*-II	EAOg36	EAHg2	GCA_025599815.1
KBV115i	Yellow-legged gull	E	ST3296	-	lambda2 (100%)	*cdtB*-II	Novel	EAHg4	GCA_025599765.1

* According Luo et al. [30]; ≥97% sequence identity, 100% sequence coverage. RSOI: Rehabilitation Center of the Swiss Ornithological Institute; GBI: Greifvogelstation Berg am Irchel; E: environment.

## Data Availability

Sequencing read data and genome assemblies have been deposited at NCBI under BioProject accession number PRJNA879956.
